# Description of A New Cryptic Species of the Papillosa Group, *Pellioditis koreana* n. sp. (Nematoda: Rhabditidae) from Korea

**DOI:** 10.2478/jofnem-2025-0056

**Published:** 2025-12-10

**Authors:** Abraham Okki Mwamula, Chang-Hwan Bae, Yi Seul Kim, Dong Woon Lee

**Affiliations:** Research Institute of Invertebrate Vector, Kyungpook National University, Sangju, 37224, Republic of Korea; Biodiversity Research Department, Species Diversity Research Division, National Institute of Biological Resources, Incheon, 22689, Republic of Korea; Department of Entomology, Kyungpook National University, Sangju, 37224, Republic of Korea

**Keywords:** DNA barcodes, morphology, morphometrics, phylogeny, taxonomy

## Abstract

*Pellioditis koreana* n. sp. was recovered from cadavers of *Protaetia brevitarsis seulensis* larvae and is herein characterized using morphometric and DNA barcode data. *P. koreana* n. sp. is characterized by its lateral fields with four lines, anterior part of the pharynx longer than posterior part, hemizonid prominent, located adjacent to the middle or posterior part of isthmus, excretory pore located anterior to basal bulb or within the anterior part of basal bulb, tail cupola-shaped, with angular sides, with a slender spike, phasmids prominent, papilla-like, flanking the cupola part, male tail region with nine pairs of genital papillae with a 1 + (1 + 1) + 2 + 1 + 3 pattern, spicules 48.0–70.5 μm long, gubernaculum less than half of spicule length long, dauer juvenile with a long tail, and tapering to a filiform posterior end. The phylogenetic relationships were reconstructed using *18S*-, *28S*-, and *ITS-rRNA* gene sequences. The phylogenies showed that *P. koreana* n. sp. is a sister species to *P. zhejiangensis*. This species represents the first record of an entomo-parasitic relationship within this predominantly gastropod-parasitic genus.

The taxonomic status of the genus *Phasmarhabditis*, Andrássy, 1976, has been controversial since its establishment. Sudhaus (1976) referred to it as the Papillosa group within the subgenus *Pellioditis* in the genus *Rhabditis* (Sudhaus and Fitch, 2001), and Sudhaus (2011) considered it to be synonymous with the genus *Pellioditis* Dougherty, 1953, with *P. pellio* Schneider, 1866 as the type species. The morphology of the two genera (*Phasmarhabditis* and *Pellioditis*) is known to be very uniform, with no clear distinguishing characteristics between them to warrant separate generic status (Huang et al., 2015; Nermuť et al., 2016a). In the recent update of the catalog of Rhabditidae by Sudhaus (2023), *Phasmarhabditis* was listed as a synonym of *Pellioditis*. This has been supported by the fact that the sequence data for the type species *P. pellio* belongs to the same clade as *Phasmarhabditis* (Sudhaus, 2023). In this study, we equally agreed with Sudhaus (2023) and treated *Phasmarhabditis* as a synonym of *Pellioditis*.

Except for *Pellioditis* (=*Phasmarhabditis*) *huizhouensis*, Huang et al., 2015, which was recovered and described from rotting leaves, members of the genus have generally been described as terrestrial facultative parasites of gastropods, though *P. hermaphrodita* (Schneider, 1859; Andrássy 1983) has been shown to reproduce in earthworms (Andrássy, 1983; Rae et al., 2006, 2009; Huang et al., 2015; Tandingan De Ley et al., 2016). Until the recent application of molecular DNA barcoding, species within the group have mostly been diagnosed using morphological characters including female tail shape and length, bursal papillae, spicule length and shape, and the presence and absence of males and morphometrics of dauer juveniles (Nermuť et al., 2016a; Ivanova and Spiridonov, 2023). During a nematological survey conducted in 2024, a cryptic population of *Pellioditis* with very close morphological resemblance to the recently described *P. zhejiangensis* (Zhang and Liu, 2020) was recovered from dark, brown-colored cadavers of *Protaetia brevitarsis seulensis* larvae. A scrutiny of morphological features and molecular DNA barcodes revealed significant differences from *P. zhejiangensis*. This species, herein designated as *Pellioditis koreana* n. sp., is described considering both morphological and molecular phylogenetic comparisons. The new species represents the first report of an entomo-parasitic relationship within this predominantly gastropod-parasitic genus.

## Materials and Methods

### Nematode population and extraction

The nematode population was extracted from dark brown-colored cadavers of *Protaetia brevitarsis seulensis* larvae recovered from soil samples taken from Gwangju, Gyeonggi-do Province, Republic of Korea. Soil samples were collected from a mountainside dominated by acutissima oak (*Quercus acutissima* Carr.) trees. The nematodes were recovered from the insect cadavers using the white trap method (White, 1927). Females, males, and dauer juveniles of *P. koreana* n. sp. were handpicked from the nematode suspension under a Nikon SMZ 1000 stereomicroscope (Nikon, Nikon Corporation Tokyo, Japan). The population was subsequently characterized based on inferences from DNA barcodes and morphometric data. Also, being a predominantly gastropod-parasitic genus, an additional preliminary test to confirm entomo-parasitic relationship was conducted on the larval stage of *Spodoptera exigua*. The susceptibility of first and second-instar larvae of *S. exigua* was tested in Petri dishes (9 cm diameter) lined with a 7 cm diameter Whatman No. 1 filter paper disk. Dauer juveniles of *P. koreana* n. sp. were applied onto the filter paper at rates of 50, 100, and 150 dauer juveniles per larva. Four larvae of each instar were gently transferred to the Petri dish and incubated at 25°C. Dauer juveniles were not added to the control, and larvae were not fed during the treatment. Larval mortality was recorded 24 h, 48 h, and 72 h after application.

### Morphological characterization

The nematodes were heat-killed and fixed with hot formalin-glycerin proportion and processed to pure glycerin according to Seinhorst (1959) as modified by De Grisse (1969). The processed specimens were mounted on permanent slides and examined under a fluorescence microscope. Photomicrographs and measurement data were taken using a Zeiss imager Z2 microscope (Carl Zeiss) fitted with Axio-vision, a material science software for research and engineering (Carl Zeiss). Line drawings were made under a drawing tube and redrawn using CorelDRAW® software version 24 (Corel Corporation Ottawa, Canada). Species diagnosis was done following original species descriptions and comparing morphometric data with the closest species of the genus.

### Molecular characterization

Genomic DNA was extracted from heat-relaxed morphometrically confirmed female and male specimens using the DNA extraction kit WizPure^™^ (Wizbiosolutions Inc. Seongnam, South Korea) according to Iwahori et al. (2000). Four gene fragments, i.e., *18S-rRNA* gene, the D2–D3 expansion segment of *28S-rRNA* gene, the partial *ITS-rRNA* gene, and the partial *COI* gene, were amplified and sequenced in this study. The two primer sets: 988F (5′-CTCAAAGATTAAGCCATGC-3′) and 1912R (5′-TTTACGGTCAGAACTAGGG-3′), and 1813F (5′-CTGCGTGAGAGGTGAAAT-3′) and 2646R (5′-GCTACCTTGTTACGACTTTT-3′) (Holterman et al., 2006) were used to amplify the nearly full-length *18S-rRNA* gene, as two partially overlapping fragments. The primer set D2A (5′-ACAAGTACCGTGAGGGAAAGTTG-3′) and D3B (5′-TCGGAAGGAACCAGCTACTA-3′) (Nunn, 1992) amplified the D2–D3 expansion segment of *28S-rRNA* gene; Vrain2F (5′-CTTTGTACACACCGCCCGTCGCT-3′) and Vrain2R (5′-TTTCACTCGCCGTTACTAAGGGAATC-3′) (Vrain et al., 1992) amplified the partial *ITS-rRNA* gene; and the partial *COI* gene was amplified using the primer set COIF1 (5′-CCTACTATGATTGGTGGTTTTGGTAATTG-3′) and COIR2 (5′-GTAGCAGCAGTAAAATAAGCACG-3′) (Kanzaki and Futai, 2002). Polymerase chain reaction (PCR) was performed with a PCR cycler (T100™, Bio-Rad Hercules, USA). The thermal cycle for the primer sets, D2A/D3B, 988F/1912R, 1813F/2646R, and COIF1/COIR2, was as described by Mwamula et al. (2023), and the thermal profile for Vrain2F/Vrain2R primer set was set as follows: initial denaturation at 95°C for 5 min, 35 cycles at 95°C for 30 s, followed by an annealing step at 53°C for 30 s; 72°C for 1 min, and finally one cycle at 72°C for 10 min. The products were purified using the QIAquick PCR Purification Kit (Qiagen Hilden, Germany) and quantified using a QuickDrop spectrophotometer (Molecular Devices San Jose, USA). The purified products were directly sequenced with the primers specified above at Macrogen Inc. (Seoul, South Korea). The new sequences were edited and submitted to the NCBI GenBank database under the accession numbers: PV031561, PV031562 (for 18S-rRNA); PV031559, PV031560 (for 28S-rRNA); PV031557, PV031558 (for ITS-rRNA); and PV031884, PV031885 (for *COI* gene).

### Phylogenetic analysis

Using the BLAST homology search program, the obtained sequences (*18S-rRNA*, *28S-rRNA*, *ITS-rRNA*, and *COI* gene) were compared with those of related species of *Pellioditis*, including comparable sequences of species from other related genera published in GenBank (Huang et al., 2015; Nermuť et al., 2016a, 2016b, 2017; Tandingan De Ley et al., 2016; Ivanova and Spiridonov, 2017, 2023; Ivanova et al., 2020, 2022; Pieterse et al., 2020; Zhang and Liu, 2020; Gorgadze et al., 2022). Multiple alignments for the selected genes (*18S-rRNA*, *28S-rRNA*, and *ITS-rRNA*) were built using ClustalX (Thompson et al., 1997). The sequences of *Oscheius citri* Tabassum et al., 2016 (MK932670), *Litoditis mediterranea* (Sudhaus, 1974) Sudhaus, 2011 (AF083020), and *Litoditis marina* (Bastian, 1865) Sudhaus, 2011 (AF083021) were used as the outgroup taxa for the *18S-rRNA* gene; *L. mediterranea* (EU195973), *Rhabditella axei* (Cobbold, 1884) Chitwood, 1933 (AY602177), and *Oscheius chongmingensis* (Zhang et al., 2008) (MT548600) were the outgroup taxa for *28S-rRNA* gene; and *Oscheius tipulae* (Lam and Webster, 1971) (JF920965), *Oscheius onirici* Torrini et al., 2015 (KX036751), and *Heterorhabditis georgiana* Nguyen et al., 2008 (KY568701) were selected as the outgroup taxa for *ITS-rRNA* gene. Bayesian inference (BI) of the phylogenies was performed using MrBayes 3.2.7 (Ronquist et al., 2012), with GTR + I + G model selected for all three datasets. BI analysis was initiated with a random starting tree and run with four chains for 1 × 10^6^ generations. The Markov chains were sampled at intervals of 100 generations. Consensus trees were generated with the 50% majority rule. The generated trees were edited using FigTree v1.4.4 software (http://tree.bio.ed.ac.uk/software/figtree/) (Rambaut, 2018). Posterior probabilities (PP) exceeding 50% are given on appropriate clades. Intraspecific and interspecific sequence variation was analyzed using PAUP* v4.0a169 (Sunderland, MA, USA: Sinauer Associates) (Swofford, 2003).

## Results

**Figure 1:**
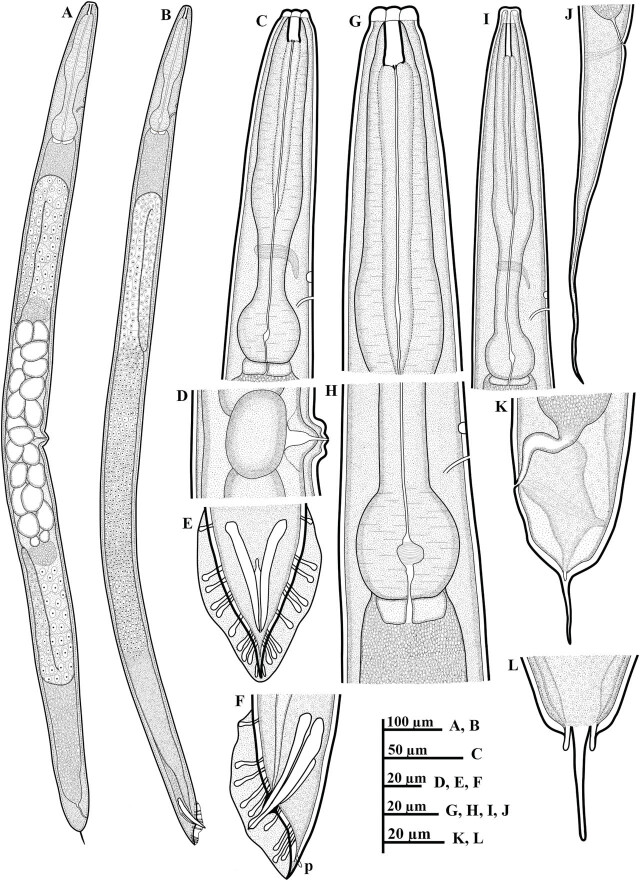
Line drawings of *P. koreana* n. sp. (A–L): (A) female whole body; (B) male whole body; (C,G) female anterior region; (D) vulval region; (E,F) posterior region of male, including copulatory apparatus and the arrangement of GP (p = phasmid); (H) basal bulb region; (I) anterior region of dauer juvenile; (J) tail region of dauer juvenile; and (K,L) female tail region including the shape of phasmids. GP, genital papillae.

**Figure 2:**
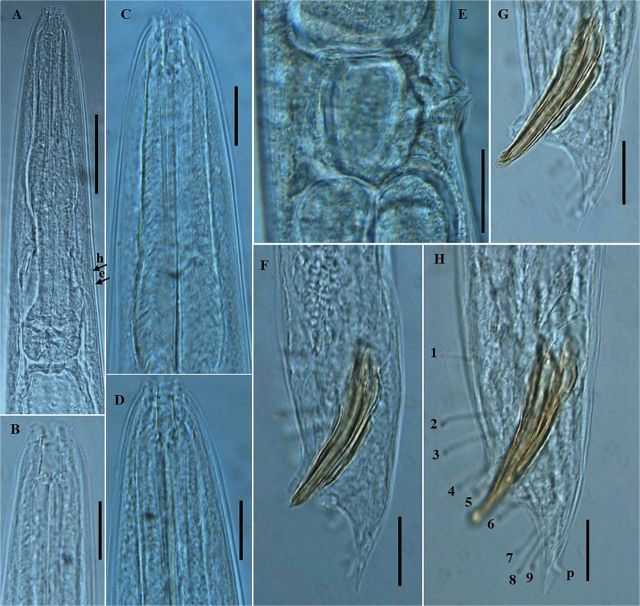
Photomicrographs of *P. koreana* n. sp. (A–H). (A–D) Female anterior region; (E) vulval region; and (F–H) posterior region of male, including copulatory apparatus and the arrangement of GP. The arrows labeled h and e indicate the position of hemizonid and excretory pore, respectively; p = phasmid (Scale bars: A = 50 μm; B–H = 20 μm). GP, genital papillae.

**Figure 3:**
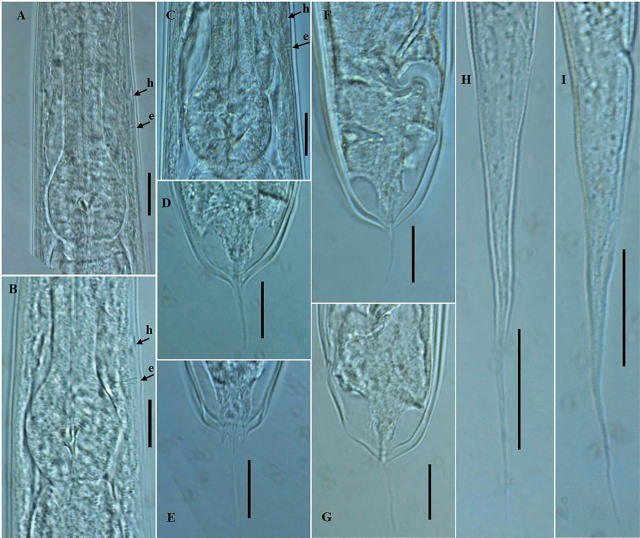
Photomicrographs of *P. koreana* n. sp. (A–I). (A–C) Posterior region of the pharynx; (D,F,G) female tail region, (E) caudal part with prominent phasmids; and (H,I) dauer juvenile tail. The arrows labeled h and e indicate the position of hemizonid and excretory pore, respectively, (scale bars: A–G = 20 μm; H,I = 30 μm).

**Figure 4:**
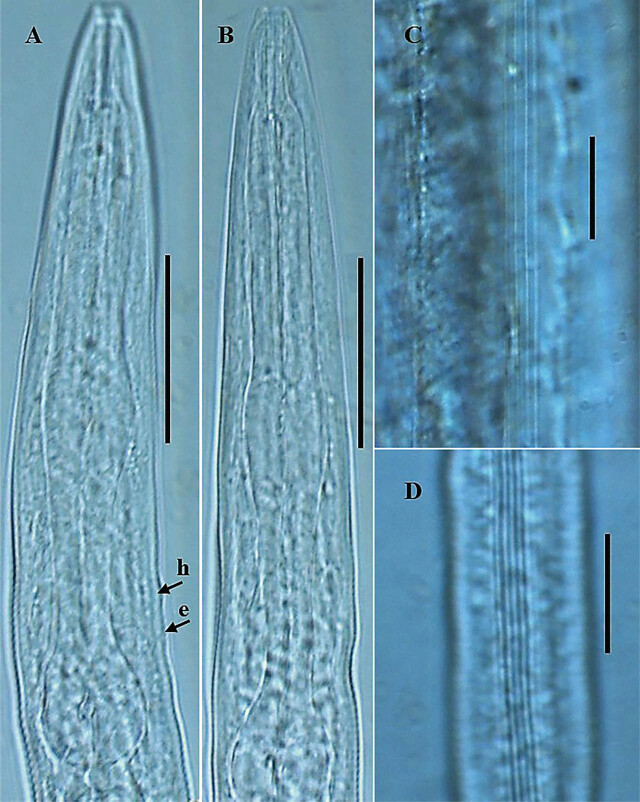
Photomicrographs of *P. koreana* n. sp. (A–D). (A,B): Dauer juvenile anterior region; (C) lateral field in female; and (D) lateral field in dauer juvenile. The arrows labeled h and e indicate the position of the hemizonid and excretory pore, respectively, (scale bars: A,B = 30 μm; C,D = 20 μm).

*Pellioditis koreana* n. sp. (Figs. 1–4)

### Measurements

See Table 1.

**Table 1:** Morphometrics of *P. koreana* n. sp. from Korea.

**Character**	**Holotype ♀**	**♀♀**	**♂♂**	**Dauer juveniles**

***n***		**20**	**20**	**20**
L	1,530.0	1,520.0 ± 116.0 (1,265.0–1,734.0)	1,181.4 ± 104.1 (998.0–1,374.0)	585.9 ± 38.2 (517.0–643.0)
a	18.1	19.7 ± 1.4 (17.2–23.7)	22.0 ± 1.0 (20.4–23.6)	22.9 ± 1.6 (20.7–26.8)
b	6.6	6.8 ± 0.5 (5.7–7.4)	6.1 ± 0.6 (4.3–6.7)	4.5 ± 0.2 (4.1–4.7)
c	29.1	27.4 ± 2.5 (23.3–31.0)	34.8 ± 2.8 (30.0–39.3)	4.9 ± 0.3 (4.4–5.4)
c'	1.3	1.4 ± 0.1 (1.2–1.6)	1.4 ± 0.1 (1.1–1.5)	7.8 ± 0.7 (6.7–9.8)
V or T	52.7	52.3 ± 1.0 (50.9–54.5)	71.1 ± 2.9 (64.8–75.2)	–
G1%	29.8	29.4 ± 2.8 (24.9–35.4)	–	–
G2%	28.7	29.2 ± 3.0 (22.3–33.8)	–	–
Lip height	6.5	5.1 ± 0.7 (4.0–6.5)	5.1 ± 0.7 (3.0–6.5)	2.0 ± 0.2 (1.5–2.5)
Lip diam.	14.0	15.6 ± 0.9 (13.5–16.5)	14.2 ± 1.0 (12.0–15.5)	5.2 ± 0.5 (4.5–6.5)
Stoma length	20.5	20.1 ± 0.5 (19.0–21.0)	19.3 ± 1.3 (16.5–21.0)	16.6 ± 0.9 (14.5–18.0)
Stoma diam.	5.5	5.8 ± 0.5 (4.5–7.0)	5.0 ± 0.7 (4.0–6.5)	–
Corpus length	134.0	130.2 ± 4.8 (120.0–138.0)	113.3 ± 5.1 (103.5–123.0)	79.4 ± 5.1 (64.0–89.0)
Metacarpal diam	29.0	30.2 ± 1.7 (26.0–34.0)	26.7 ± 1.7 (23.0–29.5)	13.7 ± 1.4 (10.5–16.0)
Isthmus length	59.5	53.3 ± 5.1 (44.5–62.5)	45.4 ± 3.6 (40.0–52.0)	31.5 ± 2.6 (24.5–35.0)
Basal bulb length	40.0	39.5 ± 1.6 (37.0–43.5)	33.9 ± 2.9 (29.5–40.5)	18.0 ± 1.3 (14.0–20.0)
Basal bulb diam.	39.5	37.2 ± 2.5 (32.5–41.5)	30.5 ± 1.9 (27.0–33.5)	16.3 ± 1.0 (14.0–18.5)
Cardia length	12.8	9.8 ± 2.9 (6.5–15.0)	7.6 ± 1.8 (6.0–12.5)	3.4 ± 0.4 (3.0–4.0)
Anterior end to nerve ring	151.5	145.5 ± 6.2 (134.0–161.0)	128.6 ± 8.0 (115.5–151.0)	90.3 ± 6.8 (72.0–107.0)
Anterior end to hemizonid	183.0	177.6 ± 9.2 (160–191.0)	155.3 ± 8.9 (136.5–169.0)	107.2 ± 8.1 (88.0–119.0)
Anterior end to Ex. pore	198.0	191.8 ± 11.4 (171.0–206.0)	170.6 ± 10.2 (148.5–185.5)	115.3 ± 7.7 (99.0–129.0)
Pharynx length	232.0	224.7 ± 7.3 (212.0–239.0)	194.8 ± 12.7 (175.5–234.5)	130.8 ± 6.2 (121.5–145.0)
Vulval body diam.	84.5	76.9 ± 7.5 (63.0–88.0)	–	–
Maximum body diam.	86.0	77.6 ± 7.8 (63.0–88.5)	53.9 ± 4.9 (44.5–63.0)	25.6 ± 2.1 (22.0–30.0)
Rectum	39.5	40.6 ± 4.0 (34.0–49.0)	–	20.8 ± 2.0 (17.0–24.0)
Anal/cloacal body diam.	42.0	41.2 ± 4.3 (34.5–50.5)	25.2 ± 3.0 (22.0–32.5)	15.4 ± 1.4 (11.5–17.5)
Tail length	52.5	55.7 ± 4.7 (46.0–62.5)	34.0 ± 2.5 (30.0–39.0)	118.9 ± 6.3 (112.0–132.0)
Tail spike length	21.5	25.3 ± 4.3 (17.0–38.0)		–
Spicules	–	–	55.2 ± 5.6 (48.0–70.5)	–
Gubernaculum			23.6 ± 1.8 (20.5–28.0)	

All measurements are in μm and in the form: average ± SD (range).

### Description

#### Female (n = 20)

Body robust, generally straight to slightly ventrally arcuate in the middle when heat-killed and fixed. Cuticle with fine annules. Lateral fields with three ridges and four lines. Lip region continuous with body, 2.2–3.8 times as wide as high, slightly flattened anteriorly with six visible lips bearing two circles of sensilla, each lip with a terminal labial papilla. Stoma 3–4 times as long as wide, with cheilostome not cuticularized. Gymnostom walls parallel, thickened. Stegostom with glottoid apparatus, metarhabdions thickened, with two wart-like denticles (Fig. 2B). Pharynx muscular, consisting of a corpus with a slightly swollen non-valvular metacarpal region, a slender isthmus, and a swollen, round, or bulbous terminal bulb with pronounced striated valvular apparatus. Cardia present, variable in length. Nerve ring encircling the slender isthmus at 61%–70% of the total pharynx length from the anterior end. Anterior part of the pharynx (procorpus + metacorpus) 1.2–1.6 times longer than the posterior part (isthmus + terminal bulb). Hemizonid clearly visible, *ca* 5 μm long, located adjacent to the middle or posterior part of the isthmus. Excretory pore prominent and with a well-cuticularized duct, located anterior to the basal bulb or within the region of the anterior portion of the basal bulb, 9.0–21.0 μm posterior to the hemizonid. The reproductive system didelphic amphidelphic, well developed with reflexed ovaries. Anterior and posterior branches of nearly similar length, filled with spherical-shaped oocytes. Spermatheca present, more visible in less gravid females, often filled with numerous sperm. Uteri of mature females filled with spherical-shaped oocytes, commonly hatching inside the body. Vagina perpendicular to body axis, its length variable, less than half of vulval body diam. in gravid females but often extending past the middle of the vulval body diam. in less gravid females. The vulva a closed transverse slit with protruding lips, located at the midbody region. The digestive system simple. Rectum prominent, wide, and curled along its length. Anus an arcuate slit. Anal body diam. 63.5%–81% of tail length. Tail with the anterior wider cupola-shaped portion, with angular sides, terminating into a moderately slender pointed spike, forming 34.5%–61.0% of the tail length. Phasmids prominent, papilla-like, flanking the base of the angular cupola part of the tail.

#### Male (n = 20)

Generally, as abundant as females. General morphology similar to that of females except for sexual characters, papillae, and a conical, pointed tail. Body straight to slightly ventrally arcuate, body generally shorter and smaller than female. Hemizonid and excretory pore prominent, positioned as in females. Testis reflexed, occupying 65%–75% of the body length. The germinal part of the testis ends in a blunt tip. Spermatogonia arranged in 2–3 rows; maturing spermatocytes located proximally in 2–3 rows. *Vas deferens* wide, filled with a large number of sperm cells. Caudal region surrounded by an open peloderan bursa, lined with nine pairs of genital papillae (GP) or bursal rays with a typical arrangement of 1 + (1 + 1) + 2 + 1 + 3 (Fig. 1E,F; 2H). In the lateral view, the first three pairs of GP (GP1, GP2, and GP3) precloacally located, with GP1 located far anteriorly from GP2 and GP3. GP2 and GP3 spaced closer to each other than to the ensuing pair (GP4 and GP5). GP4 and GP5 standing as a pair in an adcloacal position; GP6 to GP9 located post-cloacally. GP6 positioned at mid position between the preceding pair (GP4 and GP5) and the ensuing trio (GP7–GP9). GP7, GP8, and GP9 located close to each other. GP3, GP4, and GP8 are the longest, ending just short distance to the bursal edge; all other papillae shorter and do not reach the edge of the bursa. Phasmids papilla-like, located posterior to GP9 (Figs. 1E,F; 2H). Spicules paired, slightly arcuate on the ventral side, and with light swelling just before the pointed tip. Gubernaculum weakly arched, less than half (33%–48%) of spicule length long. Tail short conoid, tapering to a pointed ventrally arcuate terminus.

#### Dauer juvenile (n = 20)

General body habitus straight to slightly arcuate in the tail region when heat-killed, slender, tapering towards the head and tail end regions. Cuticle with a tessellated appearance, the outer sheath of cuticle fitted more tightly to both body ends, mouth aperture not enclosed. Lateral field with five closely spaced ridges and six lines on each side. Lip region flat, continuous with body contour, 2.3–3.5 times as wide as high. Stoma long and thin (or 7.4–13.5 times as wide as long), with cheilostom not cuticularized. Pharyngeal structure typical for the genus. Hemizonid and excretory pore visible. Hemizonid located adjacent to the middle or posterior part of the isthmus, excretory pore located anterior to the basal bulb or within the region of the anterior portion of the basal bulb, 4.0–12.0 μm posterior to hemizonid. Nerve ring encircling the slender isthmus in the anterior or mid portion. Cardia short. The rectum widens and curls along its length. Tail long, conical, and tapering to a filiform posterior end. Phasmids visible, situated 25.0–29.0 μm posterior to anus.

### Diagnosis and relationships

*Pellioditis koreana* n. sp. is characterized by its moderately robust body, 1.27–1.73 mm long, lip region 2.2–3.8 times as wide as high, lateral fields with three ridges and four lines, pharynx with rounded basal bulb, anterior part of the pharynx 1.2–1.6 times longer than posterior part, hemizonid prominent, clearly visible, located adjacent to the middle or posterior part of isthmus, excretory pore located anterior to basal bulb or within the region of the anterior portion of basal bulb, 9.0–21.0 μm posterior to hemizonid, vulva located at midbody region (51.0%–54.5%), tail cupola-shaped, with angular sides, terminating into a slender pointed spike, phasmids prominent, papilla-like, flanking the base of the angular cupola part of the tail, male caudal region surrounded by an open peloderan bursa, with nine pairs of GP with a typical arrangement of 1 + (1 + 1) + 2 + 1 + 3, spicules paired, 48.0–70.5 μm long, gubernaculum less than half of spicule length long, dauer juvenile with a long tail, conical, and tapering to a filiform posterior end.

By having a cupola-shaped tail flanked by a prominent papilla-like pointed spike, *P. koreana* n. sp. belongs to the Papillosa group and closely resembles *P. zhejiangensis* in both morphometric and molecular DNA barcode data. However, *P. koreana* n. sp. can be differentiated from *P. zhejiangensis* by its less robust body (L = 1.27–1.73 mm *vs*. 1.44–2.35 mm, and a = 17.2–23.7 *vs*. 14.1–18.8), excretory pore located anterior to basal bulb or within the region of the anterior portion of basal bulb *vs*. posterior to basal bulb, shorter cupola-shaped tail, with angular sides (46.0–62.5 μm *vs*. 60.0–83.5 μm), GP arrangement of 1 + (1 + 1) + 2 + 1 + 3 *vs*. 1 + 1 + 1 + 2 + 1 + 3, and all morphometric measurements of dauer juveniles except L (a = 20.4–23.6 *vs*. 16.1–18.9, c = 4.4–5.4 *vs*. 5.6–9.4, c’ = 6.7–9.8 *vs*. 1.1–1.5, and tail length 112–132.0 μm *vs*. 69.4–92.2 μm). *P. koreana* n. sp. is also comparable to five other members of the genus with cupola-shaped tail, including *Phasmarhabditis papillosa* (Schneider, 1866) Andrássy (1976), *Phasmarhabditis safricana* Ross et al., 2018, *P. bonaquaense* Nermuť et al., 2016b, *P. meridionalis* Ivanova and Spiridonov, 2017, and *P. huizhouensis* Huang et al., 2015. *P. koreana* n. sp. differs from *P. papillosa* by the short tail length (46.0–62.5 μm *vs*. 73.0–130.0 μm), with a short tail spike (17.0–38.0 μm *vs*. 51.0–82.0 μm), c = 23.3–31.0 *vs*. 10.9–26.3, c' = 1.2–1.6 *vs*. 1.9–3.6, prominent papilliform phasmids flanking the base of the angular cupola part of the tail *vs*. barely visible, and male GP arrangement (see Pieterse et al. (2017) for arrangement in *P. papillosa*). The new species differs from *P. safricana* by prominent papilliform phasmids *vs*. smaller, non-projecting phasmids, GP arrangement of 1 + (1 + 1) + 2 + 1 + 3 *vs*. 1 + 1 + 1 + 2/1 + 3 formulae, dauer juvenile morphometrics including head with no dorsal tooth *vs*. a single dorsal tooth present, and long tail length (112–132.0 μm *vs*. 52.0–60.0 μm, c′ = 1.2–1.6 *vs*. 8.7–9.8).

*Pellioditis koreana* n. sp. differs from *P. bonaquaense* by its less robust body (females: L = 1,265.0–1,734.0 μm *vs*. 1,878–2,626 μm, a ratio = 17.2–23.7 *vs*. 13.3–21.7; males: L = 998.0–1,374.0 μm *vs*. 1,414–2,121 μm), shorter tail length (females: 46.0–62.5 μm *vs*. 66.5–109.5 μm; males 30.0–39.0 μm *vs*. 47.0–54.5 μm), shorter spicules (48.0–70.5 μm *vs*. 68.5–86.0 μm), shorter gubernaculum (20.5–28.0 μm *vs*. 28.5–40.0 μm), GP arrangement of 1 + (1 + 1) + 2 + 1 + 3 *vs*. 1 + 1 + 1 + 2 + 1 + 3, shorter body length in dauer juveniles (517.0–643.0 μm *vs*. 808–1,050 μm), long filiform tail in dauer juveniles (112–132.0 μm) *vs*. a shorter tail with a blunt tip (71.5–102.0 μm). *P. koreana* n. sp. differs from *P. meridionalis* by the short spicules (48.0–70.5 μm *vs*. 71.0–83.0 μm), short gubernaculum (20.5–28.0 μm *vs*. 40.0–46.0 μm) prominent papilliform phasmids in females, flanking the base of the angular cupola part of the tail *vs*. less projecting and thin phasmids, tail with a moderately slender pointed spike (17.0–38.0 μm long) *vs*. a long filamentous thin spike (27.0–50.0 μm long), pharynx with rounded basal bulb *vs*. pear-shaped, vulva with protruding lips *vs*. flat lips, and shorter body length in dauer juveniles (517.0–643.0 μm *vs*. 770.0–912.0 μm). Finally, the new species differ from *P. huizhouensis* by a slender body (max body diam. 63.0–88.5 μm *vs*. 85.5–171.0 μm; a = 17.2–23.7 *vs*. 12.6–16.0), shorter angular cupola-shaped tail (46.0–62.5 μm *vs*. 81.0–105.5 μm), shorter tail spike (17.0–38.0 μm *vs*. 48.5–64.0 μm), c = 23.3–31.0 *vs*. 15.5–26.9, shorter male tail (30.0–39.0 μm *vs*. 35.5–61.5 μm), GP arrangement of 1 + (1 + 1) + 2 + 1 + 3 *vs*. 1 + 1 + 1 + 2 + 1 + 3 formulae, and short gubernaculum (20.5–28.0 μm *vs*. 30.0–41.0 μm). It is also important to note that the hemizonid has not been observed (or described) in almost all the known nominal species of the genus. However, in *P. koreana* n. sp., the hemizonid and excretory pore are prominent and clearly visible in both sexes.

### Type habitat and locality

The nematode population was extracted from brown-colored cadavers of *Protaetia brevitarsis seulensis* larvae recovered from soil samples taken from Gwangju, Gyeonggi-do Province, Republic of Korea (GPS coordinates: 37°27′10.67″N, 127°17′14.5″E).

### Type material

Holotype female, 13 females, 12 males, and 15 dauer juvenile paratypes were deposited in the National Institute of Biological Resources of Korea, and 6 females, 8 males, and 5 dauer juvenile paratypes were deposited in the Nematode Collection of Kyungpook National University (KNU), Republic of Korea.

### Etymology

*Pellioditis koreana* n. sp. was isolated and described from the Korean Peninsula region. Thus, the species epithet *koreana* is derived from the name of the country of its first description, i.e., Korea.

### Molecular characterization and phylogenetic relationships

The amplified nearly full-length *18S-rRNA*, the partial *28S-rRNA* gene, and *ITS-rRNA* gene, and the *COI* gene yielded fragments of approximately 1,700 bp, 680 bp, 990 bp, and 630 bp, respectively. The two obtained *18S-rRNA* gene partial sequences (PV031561 and PV031562) were identical with no recorded intraspecific variation. In the *18S-rRNA* gene phylogeny, *P. koreana* n. sp. sequences were grouped in a moderately supported clade with the closely homologous sequences of *P. zhejiangensis* (MT371565), *Pellioditis* sp. (MG252037), *Pellioditis thesamica* (OU753546), *Pellioditis bohemica* (KX017478, KX017479), *Angiostoma namekuji* (MF838864) and *P. huizhouensis* (KP017252), differing by 24–27 bp (2.7%–3.0%), 20–24 bp (1.3%–1.5%), 32–40 bp (2.1%–2.5%), 24–33 bp (1.5%–2.0%), 37–39 bp (2.4%) and 49–53 bp (3.2%–3.3%), respectively. The two D2–D3 sequences of *P. koreana* n. sp. (PV031559, PV031560) were also identical, with no intraspecific sequence variation (0.0%), and were also almost identical to *P. zhejiangensis* (MK937096), differing by only 3 bp (0.4%). Based on the *28S-rRNA* gene phylogeny, the sequences of *P. koreana* n. sp. were also grouped in a well-supported subclade (PP = 100%) with other sequences of *Pellioditis* spp., including *Pellioditis* sp. (PQ810652), *Pellioditis* sp. (PQ738941), *Pellioditis* sp. (PQ810653), *P. clausiliiae* (MK500248), and *P. thesamica* (OU753547), differing by 2 bp (0.3%), 14 bp (2.3%), 17 bp (2.8%), 18 bp (3.2%), and 25 bp (4.2%), respectively.

**Figure 5:**
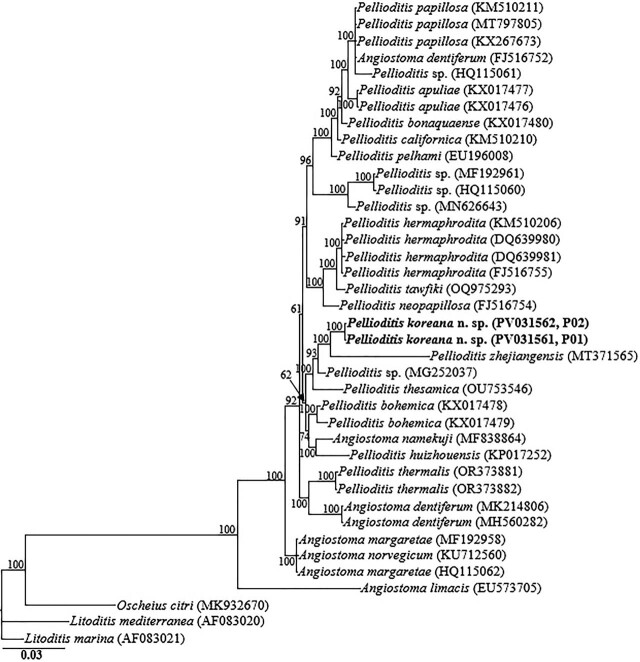
Bayesian tree inferred under the GTR + I + G model from *18S-rRNA* gene sequences of Rhabditid species. Posterior probability values exceeding 50% are given on appropriate clades. The studied population is indicated in bold text. Outgroup taxa: *O. citri*, *L. mediterranea* and *L. marina*.

**Figure 6:**
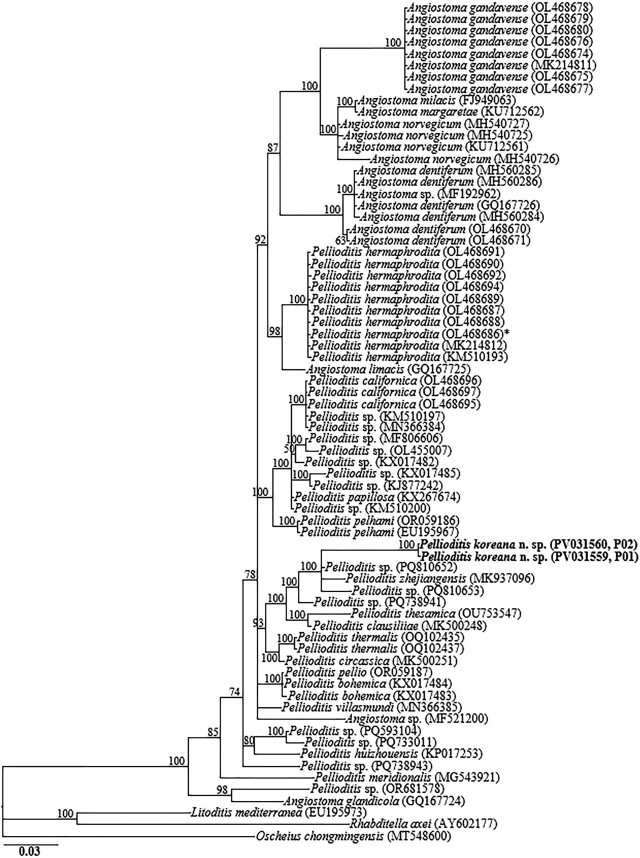
Bayesian tree inferred under the GTR + I + G model from LSU D2–D3 partial sequences of Rhabditid species. Posterior probability values exceeding 50% are given on appropriate clades. The studied population is indicated in bold text. Outgroup taxa: *L. mediterranea*, *R. axei* and *O. chongmingensis*.

**Figure 7:**
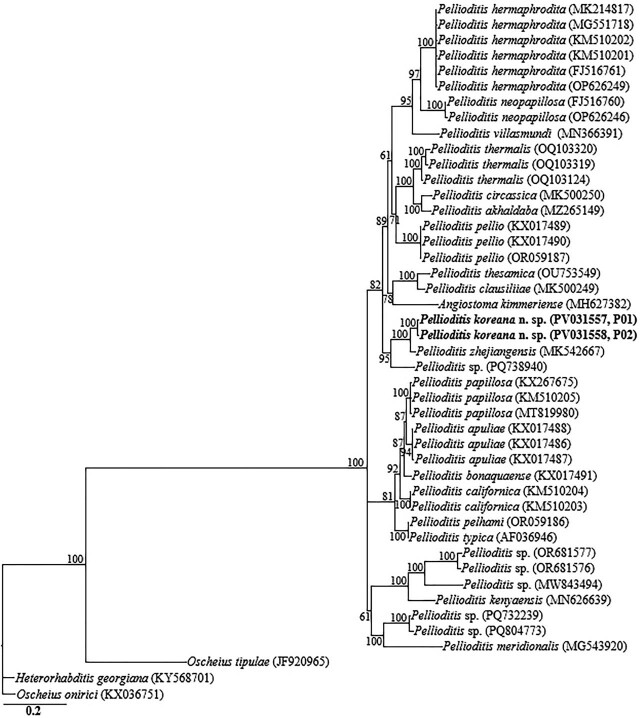
Bayesian tree inferred under the GTR + I + G model from ITS-rRNA partial sequences of Rhabditid species. Posterior probability values exceeding 50% are given on appropriate clades. The studied population is indicated in bold text. Outgroup taxa: *O. tipulae*, *O. onirici* and *H. georgiana*.

The two partial *ITS-rRNA* gene sequences of *P. koreana* n. sp. (PV031557, PV031558) were grouped in a separate well-supported clade with sequences of *P. zhejiangensis* (MK542667) and unidentified *Pellioditis* sp. (PQ738940), differing by 20 bp (2.6%) and 76 bp (8.7%), respectively. Owing to the unavailability of *COI* gene sequences for the genus in the GenBank database, the generated sequences for *P. koreana* n. sp. (PV031884, PV031885) only showed relative homology with *COI* gene sequences of *Pellioditis* sp. (MF167646), with percent identities of 89.8% based on the BLAST homology search program. Thirty-nine *18S-rRNA* gene-, 72 *28S-rRNA* gene-, and 45 *ITS-rRNA* gene sequences from member species of *Pellioditis*, and other related genera, including the newly obtained sequences and outgroup taxa, constituted the sequence dataset for phylogenetic analyses. Phylogenetic relationships, as inferred from Bayesian analysis of the dataset with the GTR + I + G substitution model, are shown in Figures 5–7.

## Discussion

Inferences from the phylogenetic analyses of the three genes, especially the partial *18S-rRNA* gene and partial *ITS-rRNA* gene sequences, suggest that *P. koreana* n. sp. is genetically distinct from the available *Pellioditis* gene sequences as represented by the Bayesian trees. The inferences suggest that *P. koreana* n. sp. is a cryptic sister species to the morphologically close *P. zhejiangensis*. *Pellioditis* is a stenomorphic genus, with very few unique and reliable morphological characters to aid species delineation due to the conservative body morphology (Nermuť et al., 2016a; Tandingan De Ley et al., 2016; Ivanova and Spiridonov, 2017; Ivanova et al., 2020). This is even exacerbated by the significant morphometric variations recorded within the same species depending on maintenance conditions of the isolates (in vivo *vs*. in vitro) (Hooper et al., 1999; Pieterse et al., 2017).

DNA barcoding is a powerful tool for nematode identification, especially for discriminating cryptic and/or very closely related species and reconstruction of phylogenetic relationships within various speciose nematode groups, including members of this genus (Pieterse et al., 2017; Zhang and Liu, 2020; Mwamula et al., 2024). Linking molecular DNA barcodes to morphological data is ideal to improve, if not solve, the taxonomic status of morphologically unresolved species (or genera) within nematode groups. However, *Pellioditis* species have been molecularly characterized using primarily ribosomal DNA fragments, especially the commonly used *18S-rRNA* and *28S-rRNA* genes. Unfortunately, the two genes appear to be less informative when comparing cryptic or closely related species of this genus. For instance, *P. californica* and *P. papillosa* display a sequence divergence of only 0.7% and 0.8% in *18S-rRNA* and *28S-rRNA* genes, respectively (Tandingan De Ley et al., 2016). In the current study, the partial *28S-rRNA* gene is almost identical to that of *P. zhejiangensis* and unidentified *Pellioditis* sp. despite the significant differences in *18S-rRNA* and *ITS-rRNA* gene sequences.

The *18S-rRNA* and *28S-rRNA* genes appear to be highly conserved within closely related species of the genus. The conservativeness of the *18S-rRNA* gene in nematode phylogeny is widely known and has been widely discussed (Blaxter et al., 1998; Holterman et al., 2009; van Megen et al., 2009; Mwamula et al., 2024), and the gene is mostly useful for reconstructing deep nematode phylogeny at higher taxonomic levels. As demonstrated by other studies (Powers et al., 2010; Palomares-Rius et al., 2017; Subbotin et al., 2018; Nguyen et al., 2019), the *COI* gene is a powerful DNA barcoding marker for nematode identification, especially in discriminating between cryptic species despite the recorded evidence for heteroplasmy, introgression, and recombination events in some nematode groups (Subbotin et al., 2020). For *Pellioditis* species, *COI* gene sequences have only been reported in a limited number of species, including *P. bonaquaense* and another unidentified population. The *COI* gene should therefore be recommended to be used as a complementary gene in the identification process of the species of this genus, as this will supplement the current generic compendia.

Inoculation of the recovered *P. koreana* n. sp. dauer juveniles onto *S. exigua* larvae confirmed the entomo-parasitic relationship between the new species and other insect pests. The new species effectively parasitized *S. exigua* larvae. *P. koreana* n. sp. parasitized 100% of first instar larvae of *S. exigua* after 24 h and 48 h at 100 and 150 dauer juveniles per larva. In the second instar larvae, the mortality reached 100% with 150 dauer juveniles per larva after 72 h. No mortality was observed in the control test. *P. koreana* n. sp. is thus a potential biocontrol agent for this commonly known polyphagous agricultural pest insect.

## References

[j_jofnem-2025-0056_ref_001] Andrássy I. (1976). Evolution as a basis for the systematization of nematodes.

[j_jofnem-2025-0056_ref_002] Andrássy I. (1983). A taxonomic review of the suborder Rhabditina (Nematoda: Secernentia).

[j_jofnem-2025-0056_ref_003] Bastian H. C. (1865). Monograph of the Anguillulidae, or Free Nematoids, Marine, Land, and Freshwater; with Descriptions of 100 New Species. The Transactions of the Linnean Society of London XXV(Part II).

[j_jofnem-2025-0056_ref_004] Blaxter M. L., De Ley P., Garey J. R., Liu L. X., Scheldeman P., Vierstraete A., Vanfleteren J. R., Mackey L. Y., Dorris M., Frisse L. M., Vida J. T. (1998). A molecular evolutionary framework for the phylum Nematoda. Nature.

[j_jofnem-2025-0056_ref_005] Chitwood B. (1933). Notes on nematode systematics and nomenclature. Journal of Parasitology.

[j_jofnem-2025-0056_ref_006] Cobbold T. S. (1884). New parasites from the horse and ass. The Veterinarian.

[j_jofnem-2025-0056_ref_007] De Grisse A. T. (1969). Redescription ou modifications de quelques techniques utilisées dans ľétude des nématodes phytoparasitaires. Mededelingen van de Faculteit Landbouwwetenschappen, Rijksuniversiteit Gent.

[j_jofnem-2025-0056_ref_008] Dougherty E. G. (1953). The genera of the subfamily Rhabditinae Micoletzky, 1922 (Nematoda). Thapar Commemoration Volume. A Collection of Articles Presented to Prof. G.S. Thapar on his 60th birthday.

[j_jofnem-2025-0056_ref_009] Gorgadze O., Troccoli A., Fanelli E., Tarasco E., De Luca F. (2022). *Phasmarhabditis thesamica* n. sp. (Nematoda: Rhabditidae), a new slug nematode from southern slope of Caucasus, Georgia. Nematology: International Journal of Fundamental and Applied Nematological Research.

[j_jofnem-2025-0056_ref_010] Holterman M., Karssen G., Van Den Elsen S., Van Megen H., Bakker J., Helder J. (2009). Small subunit rDNA-based phylogeny of the Tylenchida sheds light on relationships among some high-impact plant-parasitic nematodes and the evolution of plant feeding. Phytopathology.

[j_jofnem-2025-0056_ref_011] Holterman M., van der Wurff A., van den Elsen S., van Megen H., Bongers T., Holovachov O., Bakker J., Helder J. (2006). Phylum-wide analysis of SSUrDNA reveals deep phylogenetic relationships among nematodes and accelerated evolution toward crown clades. Molecular Biology and Evolution.

[j_jofnem-2025-0056_ref_012] Hooper D. J., Wilson M. J., Rowe J. A., Glen D. M. (1999). Some observations on the morphology and protein profiles of the slug-parasitic nematodes *Phasmarhabditis hermaphrodita* and *P. neopapillosa* (Nematoda: Rhabditidae). Nematology: International Journal of Fundamental and Applied Nematological Research.

[j_jofnem-2025-0056_ref_013] Huang R. E., Ye W., Ren X., Zhao Z. (2015). Morphological and molecular characterization of *Phasmarhabditis huizhouensis* sp. nov. (Nematoda: Rhabditidae), a new rhabditid nematode from South China. Plos One.

[j_jofnem-2025-0056_ref_014] Ivanova E. S., Clausi M., Leone D., Spiridonov S. E. (2022). *Phasmarhabditis villasmundi* sp. n. infecting land gastropods in the Nature Reserve ‘Speleological Complex Villasmundo–S. Alfio’ in Syracuse Province, Sicily. Nematology: International Journal of Fundamental and Applied Nematological Research.

[j_jofnem-2025-0056_ref_015] Ivanova E. S., Geraskina A. P., Spiridonov S. E. (2020). Two new species of *Phasmarhabditis* Andrássy, 1976 (Nematoda: Rhabditidae) associated with land snails in Northwest Caucasus, Russian Federation: Description and molecular affiliation. Nematology: International Journal of Fundamental and Applied Nematological Research.

[j_jofnem-2025-0056_ref_016] Ivanova E. S., Spiridonov S. E. (2017). *Phasmarhabditis meridionalis* sp. n. (Nematoda: Rhabditidae) from a land snail *Quantula striata* (Gastropoda: Dyakiidae) from southern Vietnam. Russian Journal of Nematology.

[j_jofnem-2025-0056_ref_017] Ivanova E. S., Spiridonov S. E. (2023). Synopsis of gastropod-associated nematodes of Ciscaucasia (Russian Federation) with the description of a new species of *Pellioditis* Dougherty, 1953 (sy. *Phasmarhabditis* Andrássy, 1976). Journal of Helminthology.

[j_jofnem-2025-0056_ref_018] Iwahori H., Kanzaki N., Futai K. (2000). A simple, polymerase chain reaction-restriction fragment length polymorphism-aided diagnosis method for pine wilt disease. Forest Pathology.

[j_jofnem-2025-0056_ref_019] Kanzaki N., Futai K. (2002). A PCR primer set for determination of phylogenetic relationships of *Bursaphelenchus* species within the xylophilus group. Nematology: International Journal of Fundamental and Applied Nematological Research.

[j_jofnem-2025-0056_ref_020] Lam A. B. Q., Webster J. M. (1971). Morphology and biology of *Panagrolaimus tipulae* n. sp. (Panagrolaimidae) and *Rhabditis* (*Rhabditella*) *tipulae* n. sp. (Rhabditidae), from leatherjacket larvae, *Tipula paludosa* (Diptera: Tipulidae). Nematologica.

[j_jofnem-2025-0056_ref_021] Mwamula A. O., Kwon O. G., Kwon C., Kim Y. S., Kim Y. H., Lee D. W. (2024). A Revision of the Phylogeny of *Helicotylenchus* Steiner, 1945 (Tylenchida: Hoplolaimidae) as Inferred from Ribosomal and Mitochondrial DNA. The Plant Pathology Journal.

[j_jofnem-2025-0056_ref_022] Mwamula A. O., Lee S. M., Jung Y. H., Lee H. W., Kim Y. S., Kim Y. H., Lee D. W. (2023). Morphological and molecular characterization of *Diplogasteroides* sp., a cryptic population of the *Haslacheri* Group (Diplogastridae), and *Parasitorhabditis terebranus* (Rhabditidae) from Korea. Journal of Nematology.

[j_jofnem-2025-0056_ref_023] Nermuť J., Půža V., Mekete T., Mráček Z. (2016b). *Phasmarhabditis bonaquaense* n. sp. (Nematoda: Rhabditidae), a new slug-parasitic nematode from the Czech Republic. Zootaxa.

[j_jofnem-2025-0056_ref_024] Nermuť J., Půža V., Mekete T., Mráček Z. (2017). *Phasmarhabditis bohemica* n. sp. (Nematoda: Rhabditidae), a slug-parasitic nematode from the Czech Republic. Nematology: International Journal of Fundamental and Applied Nematological Research.

[j_jofnem-2025-0056_ref_025] Nermuť J., Půža V., Mráček Z. (2016a). *Phasmarhabditis apuliae* n. sp. (Nematoda: Rhabditidae), a new rhabditid nematode from milacid slugs. Nematology: International Journal of Fundamental and Applied Nematological Research.

[j_jofnem-2025-0056_ref_026] Nguyen K. B., Shapiro-Ilan D. I., Mbata G. N. (2008). *Heterorhabditis georgiana* n. sp. (Rhabditida: Heterorhabditidae) from Georgia, USA. Nematology: International Journal of Fundamental and Applied Nematological Research.

[j_jofnem-2025-0056_ref_027] Nguyen V. C., Villate L., Gutierrez-Gutierrez C., Castillo P., Van Ghelder C., Plantard O., Esmenjaud D. (2019). Phylogeography of the soil-borne vector nematode *Xiphinema index* highly suggests Eastern origin and dissemination with domesticated grapevine. Scientific Reports.

[j_jofnem-2025-0056_ref_028] Nunn G. B. (1992). Nematode molecular evolution.

[j_jofnem-2025-0056_ref_029] Palomares-Rius J. E., Cantalapiedra-Navarrete C., Archidona-Yuste A., Subbotin S. A., Castillo P. (2017). The utility of mtDNA and rDNA for barcoding and phylogeny of plant-parasitic nematodes from Longidoridae (Nematoda, Enoplea). Scientific Reports.

[j_jofnem-2025-0056_ref_030] Pieterse A., Rowson B., Tiedt L., Malan A. P., Haukeland S., Ross J. L. (2020). *Phasmarhabditis kenyaensis* n. sp. (Nematoda: Rhabditidae) from the slug, Polytoxon robustum, in Kenya. Nematology: International Journal of Fundamental and Applied Nematological Research.

[j_jofnem-2025-0056_ref_031] Pieterse A., Tiedt L. R., Malan A. P., Ross J. L. (2017). First record of *Phasmarhabditis papillosa* (Nematoda: Rhabditidae) in South Africa, and its virulence against the invasive slug, *Deroceras panormitanum*. Nematology: International Journal of Fundamental and Applied Nematological Research.

[j_jofnem-2025-0056_ref_032] Powers T. O., Harris T., Higgins R., Sutton L., Powers K. S. (2010). Morphological and molecular characterization of *Discocriconemella inarata*, an endemic nematode from North American native tallgrass prairies. Journal of Nematology.

[j_jofnem-2025-0056_ref_033] Rae R. G., Robertson J. F., Wilson M. J. (2006). The chemotactic response of *Phasmarhabditis hermaphrodita* (Nematoda: Rhabditida) to cues of *Deroceras reticulatum* (Mollusca: Gastropoda). Nematology: International Journal of Fundamental and Applied Nematological Research.

[j_jofnem-2025-0056_ref_034] Rae R. G., Robertson J. F., Wilson M. J. (2009). Chemoattraction and host preference of the gastropod parasitic nematode *Phasmarhabditis hermaphrodita*. The Journal of Parasitology.

[j_jofnem-2025-0056_ref_035] Rambaut A. (2018). FigTree ver 1.4.4.

[j_jofnem-2025-0056_ref_036] Ronquist F., Teslenko M., Van Der Mark P., Ayres D. L., Darling A., Höhna S., Larget B., Liu L., Suchard M. A., Huelsenbeck J. (2012). MrBayes 3.2: Efficient Bayesian phylogenetic inference and model choice across a large model space. Systematic Biology.

[j_jofnem-2025-0056_ref_037] Ross J. L., Pieterse A., Malan A. P., Ivanova E. (2018). *Phasmarhabditis safricana* n. sp. (Nematoda: Rhabditidae), a parasite of the slug *Deroceras reticulatum* from South Africa. Zootaxa.

[j_jofnem-2025-0056_ref_038] Schneider A. (1859). Über eine Nematodenlarve und gewisse Verschiedenheiten in den Geschlechtsorganen der Nematoden. Zeitschrift für wissenschaftliche Zoologie.

[j_jofnem-2025-0056_ref_039] Schneider A. F. (1866). Monografie der Nematoden.

[j_jofnem-2025-0056_ref_040] Seinhorst J. W. (1959). A rapid method for the transfer of nematodes from fixative to anhydrous glycerin. Nematologica.

[j_jofnem-2025-0056_ref_041] Subbotin S. A., Franco J., Knoetze R., Roubtsova T. V., Bostock R. M., del Prado Vera I. C. (2020). DNA barcoding, phylogeny and phylogeography of the cyst nematode species from the genus *Globodera* (Tylenchida: Heteroderidae). Nematology: International Journal of Fundamental and Applied Nematological Research.

[j_jofnem-2025-0056_ref_042] Subbotin S. A., Toumi F., Elekçioğlu I. H., Waeyenberge L., Tanha Maafi Z. (2018). DNA barcoding, phylogeny and phylogeography of the cyst nematode species of the *Avenae* group from the genus *Heterodera* (Tylenchida: Heteroderidae). Nematology: International Journal of Fundamental and Applied Nematological Research.

[j_jofnem-2025-0056_ref_043] Sudhaus W. (1974). Nematodes (especially rhabditids) from seaweed deposits and their relationship with crustaceans.

[j_jofnem-2025-0056_ref_044] Sudhaus W. (1976). Vergleichende Untersuchungen zur Phylogenie, Systematik, Ökologie, Biologie und Ethologie der Rhabditidae (Nematoda). Zoologica (Stuttgart).

[j_jofnem-2025-0056_ref_045] Sudhaus W. (2011). Phylogenetic systematisation and catalogue of paraphyletic “Rhabditidae” (Secernentea, Nematoda). Journal of Nematode Morphology and Systematics.

[j_jofnem-2025-0056_ref_046] Sudhaus W. (2023). An update of the catalogue of paraphyletic ‘Rhabditidae’ (Nematoda) after eleven years. Soil Organisms.

[j_jofnem-2025-0056_ref_047] Sudhaus W., Fitch D. (2001). Comparative studies on the phylogeny and systematics of the Rhabditidae (Nematoda). Journal of Nematology.

[j_jofnem-2025-0056_ref_048] Swofford D. L. (2003). PAUP*: phylogenetic analysis using parsimony and other methods. version 4.0a165.

[j_jofnem-2025-0056_ref_049] Tabassum K. A., Shahina F., Nasira K., Erum Y. I. (2016). Description of six new species of *Oscheius* Andrassy, 1976 (Nematoda: Rhabditida) from Pakistan with a key and diagnostic compendium to species of the genus. Pakistan Journal of Nematology.

[j_jofnem-2025-0056_ref_050] Tandingan De Ley I., Holovachov O., Mc Donnell R. J., Bert W., Paine T., De Ley P. (2016). Description of *Phasmarhabditis californica* n. sp. and first report of *P. papillosa* (Nematoda: Rhabditidae) from invasive slugs in the USA. Nematology: International Journal of Fundamental and Applied Nematological Research.

[j_jofnem-2025-0056_ref_051] Thompson J. D., Gibson T. J., Plewniak F., Jeanmougin F., Higgins D. G. (1997). The CLUSTAL_X windows interface: Flexible strategies for multiple sequence alignment aided by quality analysis tools. Nucleic Acids Research.

[j_jofnem-2025-0056_ref_052] Torrini G., Mazza G., Carletti B., Benvenuti C., Roversi P. F., Fanelli E., Luca F. D., Troccoli A., Tarasco E. (2015). *Oscheius onirici* sp. n. (Nematoda: Rhabditidae): A new entomopathogenic nematode from an Italian cave. Zootaxa.

[j_jofnem-2025-0056_ref_053] van Megen H., van den Elsen S., Holterman M., Karssen G., Mooyman P., Bongers T., Holovachov O., Bakker J., Helder J. (2009). A phylogenetic tree of nematodes based on about 1200 full-length small subunit ribosomal DNA sequences. Nematology: International Journal of Fundamental and Applied Nematological Research.

[j_jofnem-2025-0056_ref_054] Vrain T. C., Wakarchuk D. A., Levesque A. C., Hamilton R. I. (1992). Intraspecific rDNA restriction fragment length polymorphism in the *Xiphinema americanum* group. Fundamental and Applied Nematology.

[j_jofnem-2025-0056_ref_055] White G. F. (1927). A method for obtaining infective nematode larvae from cultures. Science.

[j_jofnem-2025-0056_ref_056] Zhang C. N., Liu Q. Z. (2020). *Phasmarhabditis zhejiangensis* sp. nov. (Nematoda: Rhabditidae), a new rhabditid nematode from Zhejiang, China. Plos One.

[j_jofnem-2025-0056_ref_057] Zhang C., Liu J., Xu M., Sun J., Yang S., An X., Gao G., Lin M., Lai R., He Z., Wu Y. (2008). *Heterorhabditidoides chongmingensis* gen. nov., sp. nov. (Rhabditida: Rhabditidae), a novel member of the entomopathogenic nematodes. Journal of Invertebrate Pathology.

